# Supplementary material for: Identification of photoperiod-induced specific miRNAs in the adrenal glands of Sunite sheep (Ovis aries)
Source: Front Vet Sci. 2022 Jul 22;9:888207. doi: 10.3389/fvets.2022.888207 (PMC9354845; doi:10.3389/fvets.2022.888207)
Supplement: Supplementary file 1 [file Data_Sheet_1.ZIP › Supplementary file/miRDeep2 file.zip!/miRDeep2 file/Know_Structure/SPLP42A2/oar-mir-26a.pdf]

miRBase precursor : oar-mir-26a
Total read count : 1520181
oar-miR-26a read count : 1520041
remaining reads : 140

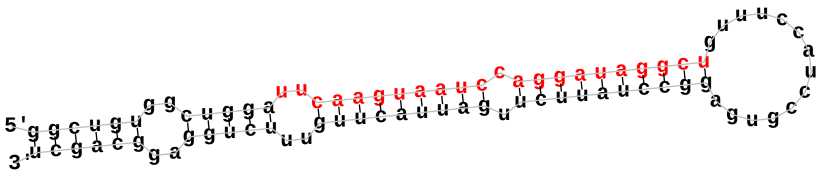

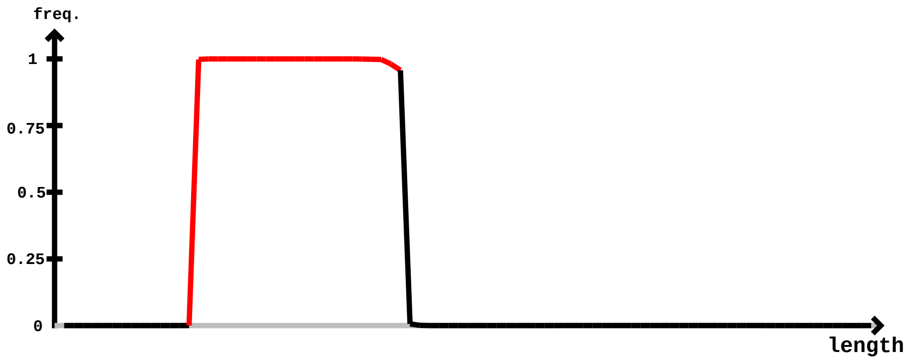


```
                oar-miR-26a
5'- ggcuguggcuggauucaaguaauccaggauaggcuguuuccauccgugaggccuauucuugauuacuuguuucuggaggcagcu  -3'   exp
    ((((((..(((((..(((((((((.((((((((((..............))))))))))).)))))))))..)))))..))))))   reads    mm     sample
    ............Guucaaguaauccaggauagg...................................................   1        1      seq
    ............auucaaguaauccaggauagg...................................................   4        0      seq
    ............auucaaguaauccaggauaggc..................................................   13       0      seq
    ............Guucaaguaauccaggauaggc..................................................   2        1      seq
    ............auucaaguaauccaggauaggcu.................................................   241      0      seq
    ............Guucaaguaauccaggauaggcu.................................................   35       1      seq
    ............auucaaguaauccaggauaggcug................................................   2        0      seq
    ............auucaaguaauccaggauaggcugu...............................................   1        0      seq
    .............uucaUguaauccaggau......................................................   1        1      seq
    .............uucaaguGauccaggau......................................................   2        1      seq
    .............uucaaguaauccaggaG......................................................   33       1      seq
    .............uAcaaguaauccaggau......................................................   2        1      seq
    .............uucaGguaauccaggau......................................................   5        1      seq
    .............uucaaguaauccaggCu......................................................   3        1      seq
    .............uucaaguaauccaggau......................................................   770      0      seq
    .............uucaaguaauccaggaA......................................................   21       1      seq
    .............uucaGguaauccaggaua.....................................................   8        1      seq
    .............uucaaguaauccaggaua.....................................................   1241     0      seq
    .............uucaaguaauccaggauag....................................................   1290     0      seq
    .............uucaaguaauccaggauaC....................................................   12       1      seq
    .............uucaaguaauccaggauagg...................................................   25436    0      seq
    .............uucaaguaauccaggauaggc..................................................   36132    0      seq
    .............uucaaguaauccaggauaggcu.................................................   1442248  0      seq
    .............uucaaguaauccaggauaggcug................................................   8039     0      seq
    .............uucaaguaauccaggauaggcugu...............................................   1390     0      seq
    .............uucaaguaauccaggauaggcuguG..............................................   8        1      seq
    .............uucaaguaauccaggauaggcuguu..............................................   19       0      seq
    .............uucaaguaauccaggauaggcuguuu.............................................   4        0      seq
    ..............ucaaguaauccaggaua.....................................................   3        0      seq
    ..............ucaaguaauccaggauag....................................................   1        0      seq
    ..............ucaaguaauccaggauagg...................................................   40       0      seq
    ..............ucaaguaauccaggauaggc..................................................   69       0      seq
    ..............ucaaguaauccaggauaggcu.................................................   2750     0      seq
    ..............ucaaguaauccaggauaggcug................................................   29       0      seq
    ..............ucaaguaauccaggauaggcugu...............................................   11       0      seq
```

oar-miR-26a

ggcuguggcuggauucaaguaauccaggauaggcuguuuccauccgugaggccuauucuugauuacuuguuucuggaggcagcu			
...............caaguaauccaggauagg...	6	0	seq
...............caaguaauccaggauaggc..	6	0	seq
...............caaguaauccaggauaggcu...	123	0	seq
...............caaguaauccaggauaggcugu...	2	0	seq
................aaguaauccaggauaggc..	2	0	seq
................aaguaauccaggauaggcu...	31	0	seq
.................aguaauccaggauaggcu...	3	0	seq
..................guaauccaggauaggcu...	2	0	seq
....ugaggccuauucuugauuacuugu..............	1	0	seq
....aggccuauucuugauuacuugu..............	4	0	seq
....aggccuauucuugauuacuuguu.............	5	0	seq
....aggccuauucuugauuacuuguuu............	1	0	seq
....ccuauucuugauuacuugu..............	3	0	seq
....ccuauucuugauuacuuguu.............	5	0	seq
....ccuauucuugauuacuuguuu............	55	0	seq
....ccuauucuugauuacuuguuuc...........	60	0	seq
....ccuauucuugauuacuuguuucu..........	2	0	seq
....cuauucuugauuacuuguuu............	2	0	seq
....cuauucuugauuacuuguuuc...........	1	0	seq
....cuauucuugauuacuuguuucu..........	1	0	seq
